# Universal Design for Learning (UDL) in simulation-based health professions education

**DOI:** 10.1186/s41077-025-00361-3

**Published:** 2025-06-12

**Authors:** Andrea J. Doyle, Michelle O’Toole, Dara Cassidy, Claire M Condron

**Affiliations:** 1 RCSI SIM Centre for Simulation Education and Research, RCSI University of Medicine and Health Sciences, Dublin, Ireland; 2 Health Professions Education Centre, RCSI University of Medicine and Health Sciences, Dublin, Ireland

**Keywords:** Universal Design for Learning; Simulation, Health professions education, Equity, Diversity, Inclusion, Accessibility

## Abstract

**Background:**

Ensuring equitable access to education is a fundamental goal in health professions training, particularly in simulation-based learning, where realistic clinical scenarios prepare learners for real-world practice. Universal Design for Learning (UDL) offers a robust framework for creating instructional strategies, materials, and environments that are accessible and effective for all learners.

**Main body:**

In this article, we provide practical guidance and actionable strategies for incorporating UDL principles into simulation-based activities. Engaging in simulation-based education requires a leap of faith and a willingness to embrace vulnerability, as learners must immerse themselves in authentic scenarios. By integrating UDL principles, educators can create a supportive environment that reduces barriers, fosters psychological safety, and ensures that all participants feel empowered to take these risks and fully engage in the learning process. This framework supports opportunities for every learner to partake in meaningful and challenging experiential learning, ultimately preparing them for successful clinical practice.

**Conclusion:**

From scenario design to debriefing techniques, this article offers insights and recommendations grounded in evidence-based practices, thereby empowering educators to optimize the effectiveness and accessibility of their simulation programs. By embracing UDL principles, educators in health professions education can create simulation experiences that cater to the diverse needs of learners, ensuring that all participants have the opportunity to thrive and succeed in their learning journeys.

## Introduction

Access to equitable education remains a persistent challenge in health professions education, including within simulation-based environments [1, 2]. Despite the potential of simulation-based education (SBE) to bridge the gap between theoretical knowledge and clinical practice, many learners still face barriers to full participation due to disability, socio-economic status, or cultural background. While simulation educators strive to design scenarios that mirror the complexity and unpredictability of real-world clinical environments [3], the diversity of learner needs is often not systematically addressed. In the absence of a cohesive framework for inclusive design, simulation activities risk privileging certain learners over others, resulting in inequitable learning experiences.

Although SBE has traditionally been guided by learner-centred approaches [4], supported by learning theories such as social cognitive theory (SCT) and communities of practice (CoP) [5–7], these pedagogies do not explicitly address accessibility or inclusion. SCT fosters autonomy and participation through observation, modelling, and feedback [5], while CoPs promote participatory learning through shared professional practice [6]. These approaches enhance engagement but may fall short in proactively accommodating diverse learner needs.

Diversity among health professions learners is multifaceted, encompassing differences in age, race, gender, socioeconomic background, disability, neurodiversity, and life circumstances such as caregiving responsibilities or part-time study [8]. Yet, many learners face systemic barriers to full participation. Learners with disabilities, for example, often encounter environments that rely on reactive accommodations, placing the burden on individuals to disclose and adapt [9]. Similarly, underrepresented minority learners, those from lower socioeconomic backgrounds, and culturally and linguistically diverse learners, continue to experience inequities in access, support, and opportunity [10–13]. While efforts to diversify health professions education have increased, they are often fragmented and insufficiently responsive to the real needs of learners [14, 15].

Universal Design for Learning (UDL), originally developed in general education, offers a proactive framework for designing flexible learning environments that anticipate learner variability [9, 14]. UDL, created by Rose and Meyer in 1984 through the Centre for Applied Special Technology (CAST, Inc.), initially aimed to support learners with additional needs using technology for personalized instruction. Over time, it has shifted to promoting inclusive design strategies that enhance learner agency and remove barriers for all. In this paper, we apply UDL principles to simulation design to support equity, diversity, inclusion, and accessibility (EDIA) in health professions education. Many of the principles underpinning UDL, such as offering multiple means of engagement, representation, and expression, align with simulation pedagogy, yet they have not been widely applied in this context. This represents a significant gap in both practice and research.

This article introduces UDL as a relevant and underutilised framework for enhancing equity and accessibility in SBE. We aim to:

1. Provide a pragmatic overview of UDL principles and how they apply to simulation;
2. Identify parallels between UDL and existing practices in SBE;
3. Offer realistic, actionable guidance for inclusive simulation design, summarised as practical tips throughout the article.

We argue that UDL offers a valuable lens through which simulation educators can amplify accessibility across the simulation continuum—design, delivery, and debriefing—and we hope to motivate meaningful change toward more inclusive educational practices.

### Diversity and inclusion in health professions learners

Approximately 1.3 billion people experience disability, representing 16% of the world population [9]. The World Health Organisation’s (WHO) conceptualization of disability has shifted from the social consensus between normalcy and dysfunction to a more relational and individualistic understanding of embodied impairment and the environmental constraints placed on people with disabilities [14]. In 2021, the WHO adopted Resolution 74.8 to make the health sector more inclusive by addressing the barriers facing persons with disabilities when accessing health services, including the disability-sensitive education of health workers [15]. Efforts to diversify health professions education and adopt an inclusive approach to learning have historically been tokenistic and disconnected from the true needs of learners [14]. Progress towards increasing the percentage of people with disabilities among the health professions population will require creativity and dedication at every turn. This will necessitate a change in the status quo in health professions education. There is a need to move away from *reasonable accommodations*, which often places the onus on learners to disclose their disability, potentially resulting in further stigmatisation [16]. Instead, we should aim to design education to be inclusive for all, to diversify learner populations, proactively seeking diversity as a strength, rather than a deficit that requires reactive adaptations [8]. UDL provides a framework that can support the redesign of health professions curricula to facilitate access and participation, opening the door for all learners to engage in relevant, demanding learning opportunities in preparation for real world situations [10].

What do we mean by diversity in the context of learners in health professions education? Diversity in health professions education is multifaceted, encompassing various aspects of learners’ backgrounds and circumstances. Key factors include age, race, religion, socioeconomic status, gender, neurodiversity, health conditions or impairments, and situational differences such as employment, caregiving responsibilities, part-time enrolment, and the use of online learning modalities [11]. Underrepresented minority learners often encounter significant barriers in admissions [17], due to systemic biases and a limited applicant pool [12, 13, 18]. Culturally and linguistically diverse learners face unique challenges in clinical placements, including language barriers and cultural differences, which can hinder their learning experiences [19]. Enhancing racial and ethnic diversity in health professions is essential for providing culturally competent care and addressing healthcare inequalities [13, 19]. Learners from higher socioeconomic backgrounds, particularly those with parents in top income brackets or healthcare professions, have higher odds of admission to health professions programs [13]. This underscores the need for more equitable admissions processes. Although gender diversity has improved, with women now being fully represented in medical schools, disparities still persist in certain specialties and faculty positions [20]. Continued efforts are necessary to address these gender-based disparities. Learners with neurodiversity, including those with dyslexia, Attention-deficit/hyperactivity disorder (ADHD), autism, and other conditions, face significant challenges in higher education. These challenges often stem from a lack of adequate support and fear of stigmatization [21–23]. Providing appropriate accommodations and fostering an inclusive environment are critical for their success.

### Universal Design for Learning (UDL)

**Fig. 1:**
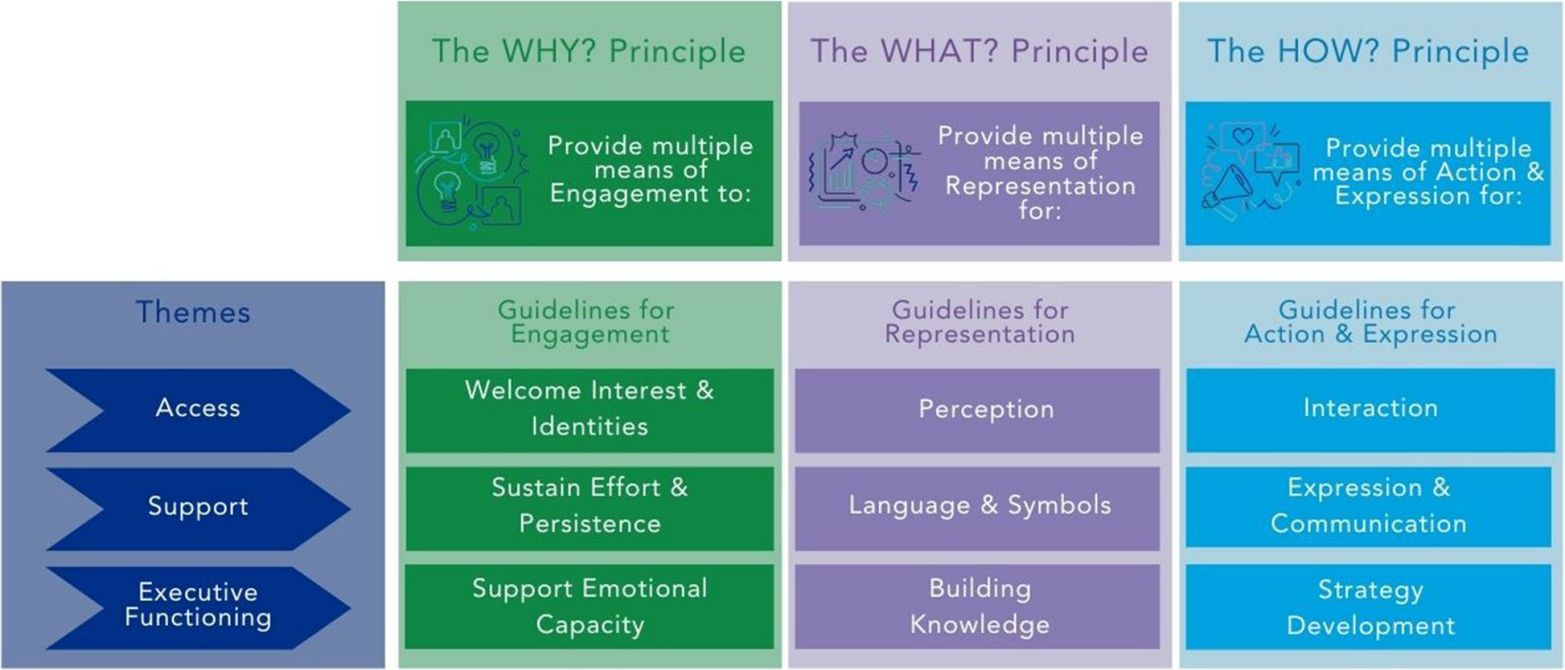
UDL principles and guidelines, adapted from CAST [10]

Universal Design for Learning (UDL) is an educational framework informed by research in neuroscience and learning sciences, that aims to create adaptable learning environments capable of addressing individual variations in learning needs [24]. In its initial iteration, UDL sought to transform the way learners with additional needs received instruction, employing technology to assist teachers in personalizing teaching methods for learners with learning disabilities. To achieve this goal, Rose and Meyer created The Centre for Applied Specialized Technology (CAST, Inc.) in 1984. Since then, UDL has evolved from a focus on addressing individual learning requirements to a focus on educational design strategies that support learner agency, while seeking to remove barriers to learning for all. While there are now legislative equality requirements to prevent ableist discrimination and require reasonable accommodations, UDL promotes the educational design principle that what is *necessary for some, benefits everyone*. UDL is described by three principles, nine guidelines and 36 considerations [10], which offer a framework that promotes the creation of instructional materials, activities, and environments that are accessible and beneficial to all learners, regardless of their diverse backgrounds, abilities, or learning preferences. Figure 1 depicts an accessible summary of the UDL principles, guidelines and themes. In the UDL framework, the principles address the ‘Why’ or the motivation for learning, the ‘What’ or the content of educational materials and importantly the way it is represented, and the ‘How’, or the ways in which learners interact with material [10]. Each principle is broken down into three guidelines, focused on operationalising the specific principle. These guidelines are further broken down into *considerations* [10] specific, actionable strategies nested under the nine guidelines. They offer detailed suggestions to help educators design inclusive, flexible learning environments that accommodate the diverse needs of all learners. Central to this are equity, diversity, inclusivity, and accessibility (EDIA) initiatives.

#### Core principles and cross-cutting themes of the UDL framework

The foundation of UDL philosophy lies in the core principles; that there are numerous ways to present knowledge (representation principle), diverse methods for learners to showcase their understanding (action and expression principle), and multiple approaches to engage learners in the learning process (engagement principle) (see Fig. 1). The framework includes cross-cutting themes of access, support and executive functioning, that relate to an individual’s progression, and help scaffold the UDL framework in ensuring that all learners have the opportunity to succeed:

#### Access

This refers to ensuring learners can access learning opportunities, materials, and the environment. The “access” phase of the guidelines emphasizes the need to remove barriers so that learners of varying abilities can participate from the outset. It's about providing equitable access to the curriculum by offering multiple means of engagement (motivating learners), representation (how information is presented), and action/expression (how learners demonstrate understanding).

#### Support

Support can be interpreted as the means by which educators help learners as they progress through different stages of learning. This includes providing scaffolding, tools, and resources that aid understanding, retention, and skills development, helping them move beyond basic access to a deeper interaction with learning materials and experiences.

#### Executive functioning

This term relates to a learner’s ability to set goals, plan, organize, strategize, and manage their learning. In the UDL framework, providing support for executive functioning involves helping learners with planning and goal setting, managing information and resources, and guiding the development of self-monitoring and reflection skills. It addresses how learners regulate their learning, ensuring they have the tools to take ownership and self-direct.

Health professions education is strictly regulated and standardised to support learners to achieve the intended learning objectives and become competent practitioners for the populations they serve. While some aspects of educational design cannot be altered without significant curricular overhaul and re-accreditation processes, adopting a UDL lens can help identify small-scale, achievable changes that may be impactful.

While SBE is often reported to be favoured over traditional didactic teaching [25], it demands a transition from private learning practices to active participation in SBE and can be challenging for learners; whereupon learners have reported negative interactions and feeling unsafe [26], and others have also experienced anxiety [27]. The impact of SBE on learner’s confidence and anxiety is multifaceted; while some learners report it can be more stressful than real-life emergencies, data suggests SBE generally improves learners'confidence and perceived self-efficacy [27]. The transition to SBE introduces learners to peer scrutiny and heightens performance pressure, creating various stressors that may affect their learning experience and overall well-being. The discomfort some learners experience when shifting from private learning to active, public participation highlights the diverse needs and preferences of learners. UDL’s emphasis on providing multiple means of engagement, representation, and expression helps mitigate performance anxiety, making active learning experiences such as SBE more accessible, supportive, and effective for diverse learners [10, 28].

## Applying the UDL framework to simulation

UDL promotes the creation of flexible and adaptable interventions tailored to individual learner needs. It reflects an anticipatory design approach, where learning environments, materials, or systems are created in a way that predicts and addresses potential barriers before learners encounter them, akin to User Experience (UX) design in the technology sector [29]. Anticipatory design involves streamlining processes and addressing user needs proactively, often before they are explicitly stated, for example, incorporating captions or transcripts in all educational videos by default, rather than waiting for a learner to request them. Similarly, UDL aims to create educational environments that offer equal learning opportunities for all learners, regardless of their diverse backgrounds and capabilities.

### Simulation design

#### Co-design

Engaging learners in the design process supports the integration of UDL principles into curricula, promotes learner engagement, and facilitates anticipatory design [30]. By assuming that learner groups will be diverse from the outset, we can mitigate ableist design decisions and promote equity and neuro-inclusion. Focusing on the why of learning, simulation educators can engage learners or their representatives directly in the scenario design process, helping to prevent tokenistic or performative engagement in EDIA initiatives [31]. Co-design creates an opportunity to better represent learners’ goals and objectives in scenarios, potentially enhancing the relevance and authenticity of these scenarios, while simultaneously supporting learners’ engagement and their motivation to learn. In health professions simulation, participatory design activities, in which learners co-construct simulation scenarios, have reported rich, learner-driven experiences that promoted reflection and supported identification of learning goals [32, 33].

#### Clarity of goals

Within a UDL context, well-defined learning objectives are critical for guiding learners’ goal setting and enhancing their capacity to monitor progress. Such clarity is a cornerstone of curriculum design, often determining the educational content and pedagogical approach [34]. In simulation, this is often done extremely well, and scenarios routinely have specific and measurable learning objectives to prepare learners for patient and professional interactions. This supports the transfer of their learning from the simulated environments to their future clinical practice. To enhance this to align with UDL guidelines, the learning objectives could be made available in advance of the session, to facilitate learner orientation and reduce anxiety associated with uncertainty [35].

#### Diversity in representation

There is a movement to diversify and “decolonise” health professions education, to incorporate more culturally responsive and equity focussed curricula [36–38]. A UDL approach can facilitate the extension of this to educational content. In simulation, scenario materials can be purposefully designed to reflect diversity in gender roles, simulated patient identities, patient histories and physical and neurological disabilities [39–43]. The need for improved racial diversity in simulators and manikins in health professions education has been reported [44], noting low representation for communities of colour, including Black, Hispanic, and Asian populations.

This focus on diversity can also be reflected in the ways that key information is presented to learners. It should be recognised that there is not one means of representation that will accommodate everyone. It can, therefore, be useful to provide information to learners ahead of their simulated training in flexible formats. For example, video or audio content pertaining to the patient case or a podcast, can be presented to allow user control of playback speed and the inclusion of closed captions for those who may have an auditory impairment, or are learning in a different language to their mother tongue. While printed and physical materials, such as medical reports, patient charts, or diagnostic information, are often inflexible, various features can be modified for inclusivity, including font, text size, and the contrast between text and surrounding content, as well as the size of images, graphs of figures and the content they contain [45]. Including learners in this aspect of content design for simulated scenarios will help to represent the information in a more practically inclusive way.

#### Pre-brief

In SBE, pre-briefing helps to prepare learners for the learning activities and reduces learner anxiety [35]. The pre-brief is an opportunity to establish psychological safety; to create a supportive social environment where learners can feel safe to try, fail and learn [46]. Simulation is a participatory educational technique, and it is important to acknowledge the discomfort and stress that this can exert on learners [47]. This discomfort can be particularly overwhelming for learners who may find it challenging to self-regulate [48]. However, by engaging in simulations, learners may have the opportunity to practice and hone their coping skills and develop internal controls to manage and direct emotional responses to external events. Bringing a UDL lens to the planning and design of the pre-brief can help promote a psychologically safe environment to help all learners get the most out of the simulation session. By emphasizing multiple means of engagement, representation, and expression, UDL may help address performance anxiety, making active learning experiences like SBE more inclusive, supportive, and effective for diverse learners. UDL’s focus on inclusivity and considerations relating to goal setting, addressing biases, and maximizing transfer and generalization, align well with Somerville et al.’s tips for designing, implementing, and steering a pre-brief to promote psychological safety [35].

The basic assumption, confidentiality, and the fiction contract.

“We believe that everyone participating in simulation activities is intelligent, capable, cares about doing their best and wants to improve.”

The Basic Assumption (Centre for Medical Simulation 2022).

The Basic Assumption is inherently inclusive and is a prime example of where a UDL perspective is already a feature of simulation. Recognising and appreciating diversity, it establishes the precedent that simulation is a process through which learners can improve, and reminds the simulation community to *assume the best*, from everyone they encounter. This assumption coexists with the understanding of confidentiality, which is an essential aspect of psychological safety in simulation. However, simulation is not an entirely controlled environment; learners do not enter or exit a simulated scenario and debriefing as blank slates. The primary objective of experiential learning is to facilitate the transfer of performance skills from the simulation to real-world applications [49]. While the specific details of individuals’ performance and scenario outcomes should remain confidential, the learnings from simulation interventions are open for discussion and should be translated into future clinical practice [35]. This requires learners to feel safe to share their perceptions, feelings and insights following simulation, and for some learners this can be challenging. Simulation is undoubtedly an experiential approach to learning, but it is also social. Learners learn not only by doing, but also by observing and interacting with their peers. Confidentiality is important to the social and esteem needs of learners, and this resonates with the principles of neuro-inclusion [50]. Studies have reported that some learners find social functioning overwhelming and describe difficulties with self-expression, which can lead to isolation and loneliness [51]. SBE can support the UDL Engagement principle (Fig. 1) to foster collaboration, interdependence, and collective learning, providing psychologically safe communities of learning that encourage and support peer-interaction.

The fiction contract also relates to psychological safety as educators ask learners to suspend disbelief and act as if the simulation is real, so that they interact in the simulated environment as they would behave in clinical practice [52]. This requires a social agreement, asking learners to be motivated and somewhat vulnerable to engage in the scenario. Social interactions and social functioning can be challenging for neuro-diverse learners, with a higher risk of experiencing isolation and poor self-esteem [48]. Educators must therefore facilitate psychological safety from the pre-brief, to avoid perceived potential harm and mitigate learner shame [35]. We implore simulationists to continue to promote the basic assumption, and to emphasise that mistakes are welcomed and seen as positive learning opportunities. UDL’s cross-cutting ‘Support’ theme (Fig. 1), includes considerations that may help foster psychological safety.

#### Orientation to the simulated environment

Simulation offers a safe environment to develop, practice and master skills away from the high-stakes clinical environment, however, simulated scenarios and training interventions can themselves induce stress in learners [53]. In high-fidelity simulated clinical environments, simulation provides learners with the opportunity to interact with equipment and tools they will be expected to use in the clinical setting. Early exposure to these objects and artefacts enables learners to become familiar thus reducing anxiety. It also affords an opportunity for learners to identify additional needs they may have to complete tasks, i.e. text to audio, a joystick or an adapted keyboard. Learners might need a period to adjust to the simulation setting, and there are reported benefits, including reduction in anxiety and increased confidence, associated with pre-simulation orientation [54]. Planning orientation time before simulations begin offers learners the chance to familiarise themselves with the environment, personnel and equipment reducing uncertainty [35]. Additionally, for those learners with diverse needs, pre-simulation orientation may help to plan access routes, space for self-regulation and mitigate sensory overload, all of which could negatively impact learner performance [51]. UDL considerations *Address biases, threats, and distractions* and *Optimize access to accessible materials and assistive and accessible technologies and tools* [10] provide useful guidance in this regard.

#### Clarify content and answer questions

Academic functioning can be demanding for learners, and this is addressed through some of the UDL guidelines in the framework (see Fig. 1) [10]. Providing learners with more options for comprehension can help translate information into knowledge. Comprehension can be a challenge for some learners and therefore signposting information, expectations and instructions as part of the orientation can support this process [51]. Clarifying the goals of the simulation in advance helps learners to link concepts and revisit key ideas. Providing background information in multiple forms prior to learning activities could help to activate prior knowledge during simulations and to bridge core concepts [10, 55]. Additionally, this is an opportunity to provide clarity around key terminology, abbreviations and acronyms, which are frequently employed in health professions education.

Table 1 presents some simple examples of how the UDL principles could support the design of more inclusive SBE.

**Table 1:** UDL principles in the design of SBE activities

Design consideration	SBE example	UDL principles
Accessible spaces	Designing simulation rooms with adjustable-height beds, wide doorways, and unobstructed layouts to accommodate wheelchair users without requiring special arrangements	Supports the representation and engagement principles by ensuring all learners can physically access and engage with learning environments without needing to self-advocate for changes. This aligns with providing multiple means of access from the outset
Flexible briefing formats	Providing pre-simulation materials in multiple formats (e.g. written, audio, video) so learners with different processing preferences or needs can engage effectively, without having to disclose a learning difficulty	Aligns with the Representation principle—offering multiple ways to acquire information—and supports learners with different sensory, cognitive, or linguistic needs
Multimodal assessment options	Allowing learners to demonstrate understanding through oral explanation, written reflection, or practical demonstration, rather than a single mandated method	Directly addresses the action and expression principle by allowing learners to demonstrate understanding in diverse ways. This supports learner agency and reduces barriers to assessment
Built-in quiet zones	Including designated low-stimulation areas for learners who may need a break from sensory input during high-stress scenarios, without requiring prior request	Falls under the Engagement principle by considering emotional regulation and promoting sustained effort and persistence. It also supports executive functioning needs by facilitating self-regulation and reducing anxiety triggers
Neutral and inclusive case design	Creating scenarios that avoid assumptions about gender, ethnicity, or cultural background of patients or learners, allowing everyone to see themselves reflected and respected in the content	Supports engagement by fostering a sense of belonging and relevance, especially for learners from underrepresented or marginalised groups. It also links to the representation principle by ensuring culturally diverse perspectives are included and respected

### Simulation delivery

#### Roles and participation

In SBE, action and engagement, key UDL principles, are a prerequisite; learners are expected to be immersed in simulated scenarios. The immersive nature of simulation encourages participation and practice. Although SBE is inherently experiential and emphasizes active participation, the role of observer also provides benefits [56]. Equivalent levels of non-technical performance between observers and active participants have been reported, as well as a reduction in stress [57]. An observer role in simulation could offer a supportive pathway for learners who might initially be hesitant to participate, to eventually become actively involved in SBE roles. Additionally, by observing simulation interactions, learners can provide feedback to their peers and provide opportunities to reflect on their strengths and challenges, and those of their peers [56].

#### Scaffolding learning

Integrating prompts or nudges within the simulation can provide additional information or guidance for participants to aid their comprehension. This feedback can come in various forms, for instance in virtual reality simulations, pop-up messages, audio cues, or visual indicators and can provide guidance for learners. In-person, in a simulated clinical environment, an embedded participant can provide feedback, cues and guidance to learners [58]. Embedded participants play the role of a healthcare worker, a family member or a parent, often shaping the course and emotional intensity of a simulation [59]. These cues, through computer generated prompts or from an embedded participant, help to scaffold a learner as they practice and develop independence, building fluencies in their actions [10]. Learning objectives can be staged and scaffolded to support skill development, and repeated practice through part task training [60] can help learners to build fluencies, which is an important guideline from the UDL framework for action and expression [10]. Simulation facilitates contextualisation of information and knowledge, and during simulated interventions learners often re-encounter material with which they have previously engaged. This opportunity for integration supports learners to develop the skills and confidence to build from isolated task practice, and then tasks are spiralled to participation in whole-task simulations. Whole-task training is more complex and challenging, and representative of clinical practice, than part-task simulated training [61], e.g. gaining the motor skills for a kidney biopsy on a model-based task trainer, followed by immersive training to gain the critical thinking and decision-making skills required to perform the biopsy at the bedside.

Simulation can be overwhelming and the realism or simulation fidelity which facilitates emotional engagement, is often accompanied by emotional stress [53, 54]. While many believe that stress can promote learning, others report that stress generates an unsafe learning environment, negatively impacting learning and memory recall [26, 62]. Individual responses and coping mechanisms in such situations may vary, and therefore so too will an individual’s ability to self-regulate. UDL welcomes opportunities to provide scaffolds to support learners to develop their intrinsic abilities to regulate their emotions [10]. The UDL principles include considerations that can help simulationists proactively design strategies for emotional regulation into the simulation experience [10]. Acknowledging the stress before a simulation encounter and offering strategies to manage and self-regulate through goal setting and self-reflection, reportedly increases learners'performance [63]. Allowing learners to take a break and restart simulations if the environment becomes too intense has proven an effective strategy for managing stress. This approach, which includes time for debriefing, resulted in learners feeling less anxious and having greater clarity about the situation, while maintaining the realism of the simulation [64].

Table 2 illustrates concrete examples of how UDL principles can be applied across high-fidelity manikin, simulated patient, and virtual reality (VR)-based simulations, demonstrating how inclusive design can enhance learner engagement, flexibility, and performance across diverse educational contexts.

**Table 2:**
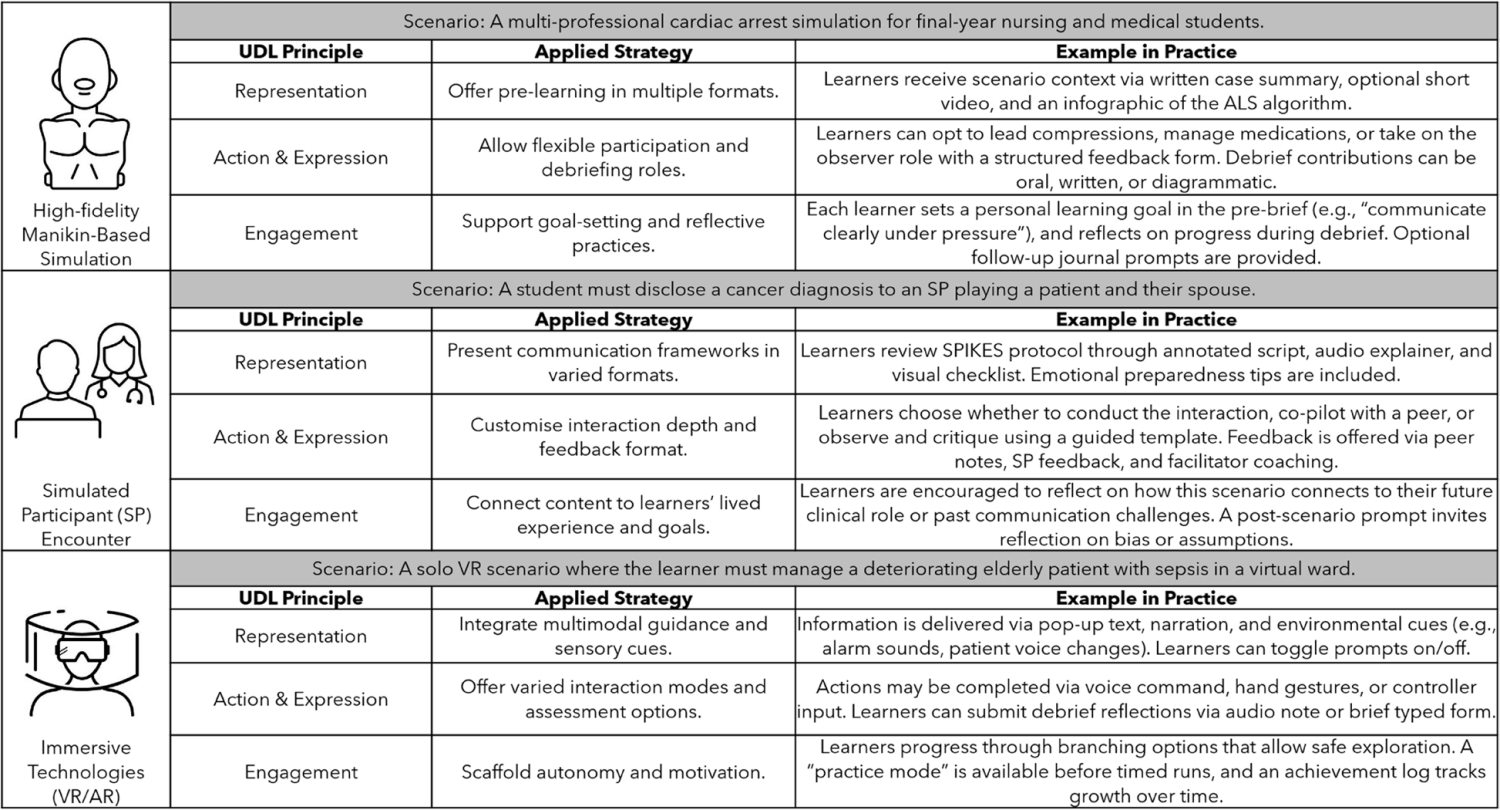
Operationalising UDL access across simulation modalities

### Simulation debriefing

The objective of UDL is to prepare learners with the necessary skills to become (1) purposeful and motivated (engagement principle), (2) resourceful and knowledgeable (representation principle), and (3) strategic and goal directed (action and expression principle) (see Fig. 1) [10]. Through reflective practice, learners can evaluate their learning needs, establish personal goals, and track their progress. Reflecting on the simulated event offers an opportunity to identify positive behaviours and learner performance as well as aiding in identifying and addressing gaps in knowledge, performance and skills [65]. Debriefing serves as a guided group reflection within an experiential learning cycle [65]. There are a range of techniques for debriefing, sometimes occurring during and throughout a simulation, or after the simulation has ended. Successful debriefing experiences will include motivated and engaged learners as well as experienced and prepared facilitators [66]. Debriefings require learners to be vulnerable and open to discussions about their performance and behaviours during experiential learning activities, and psychological safety is key to a successful open reflective process [67].

Psychological safety is critical throughout the process of simulation and is not a static concept; it needs to be maintained and strategies originating at pre-briefing stage should be carried right through to debriefing [35, 46, 47]. Creating this “safe container” [47] reduces distress, distrust and reluctance to engage, and potentially eases the stress and tension experienced by learners who have challenges with social functioning and self-expression [51]. The UDL considerations within strategy development (Fig. 1) can offer valuable guidance here. Tools, checklists, and rubrics such as PEARLS (Promoting Excellence And Reflective Learning in Simulation) [65] can be incorporated into the debrief as a structure for learners to review their own performance and generate self-directed goals. Many simulation centres are equipped with audio and video recording technologies that facilitate learners to review their simulated interactions. However, this should be carefully balanced through supportive debriefing practices, as the use of such recording technologies could act as a stressor for learners, who may feel heightened anxiety knowing their performance is being captured and subject to review—for example, making visible mistakes, peer judgment, or fear of negative evaluation. This heightened stress can interfere with cognitive processes such as planning, focus, information recall, and task management, which are key components of executive functioning. These challenges may be particularly pronounced in neurodiverse learners, for whom executive functioning deficits are common. Supporting these learners to develop self-reflection skills is therefore essential [68]. Structured debriefing and feedback models can provide the tools needed not only to process simulated learning experiences but also to strengthen everyday strategic thinking. By embedding these strategies into debriefing conversations, educators create opportunities for learners to explore different approaches, reduce cognitive load, and benefit from shared peer insights.

### Navigating the practical challenges of UDL in simulation-based education

UDL offers a valuable framework for enhancing inclusivity in SBE, but its implementation can be challenging. Clinical simulation is often shaped by strict objectives, protocols, and standards focused on patient safety and professional competence, which can appear at odds with UDL’s emphasis on flexibility and personalisation. However, with intentional design, these approaches can coexist, maintaining fidelity while embedding flexibility where appropriate.

A key tension is balancing UDL-informed customisation with the logistical demands of large cohorts. Limited time, staff, and resources can constrain efforts to diversify engagement or assessment. Additionally, institutional norms may favour standardisation over innovation, and educators may lack the training or support needed to implement inclusive design effectively. The UDL community acknowledge this and propose the “Plus One” approach as a pragmatic strategy [69]. “Plus One” encourages, small incremental changes to curricula as an introduction to the UDL principles defined by CAST [10]. By changing one small element in or design of simulated interactions, potentially huge gains could be made in terms of EDIA implementation across SBE and health professions education in general.

Throughout this article we have suggested practical tips for simulation educators adopting UDL principles when designing or redesigning simulated scenarios. Many higher education institutions have incorporated anti-discriminatory legislation and provide designated support services for learners and staff [21]. These services often include staff training to support learners that may have additional requirements and specific needs, offering an opportunity to embed UDL principles in their content and design. Other approaches to implementing UDL principles across a team, department or university include creating communities of practice, forming working groups, developing and adopting policies and guidelines, and providing training and professional development for staff [70].

**Fig. 2:**
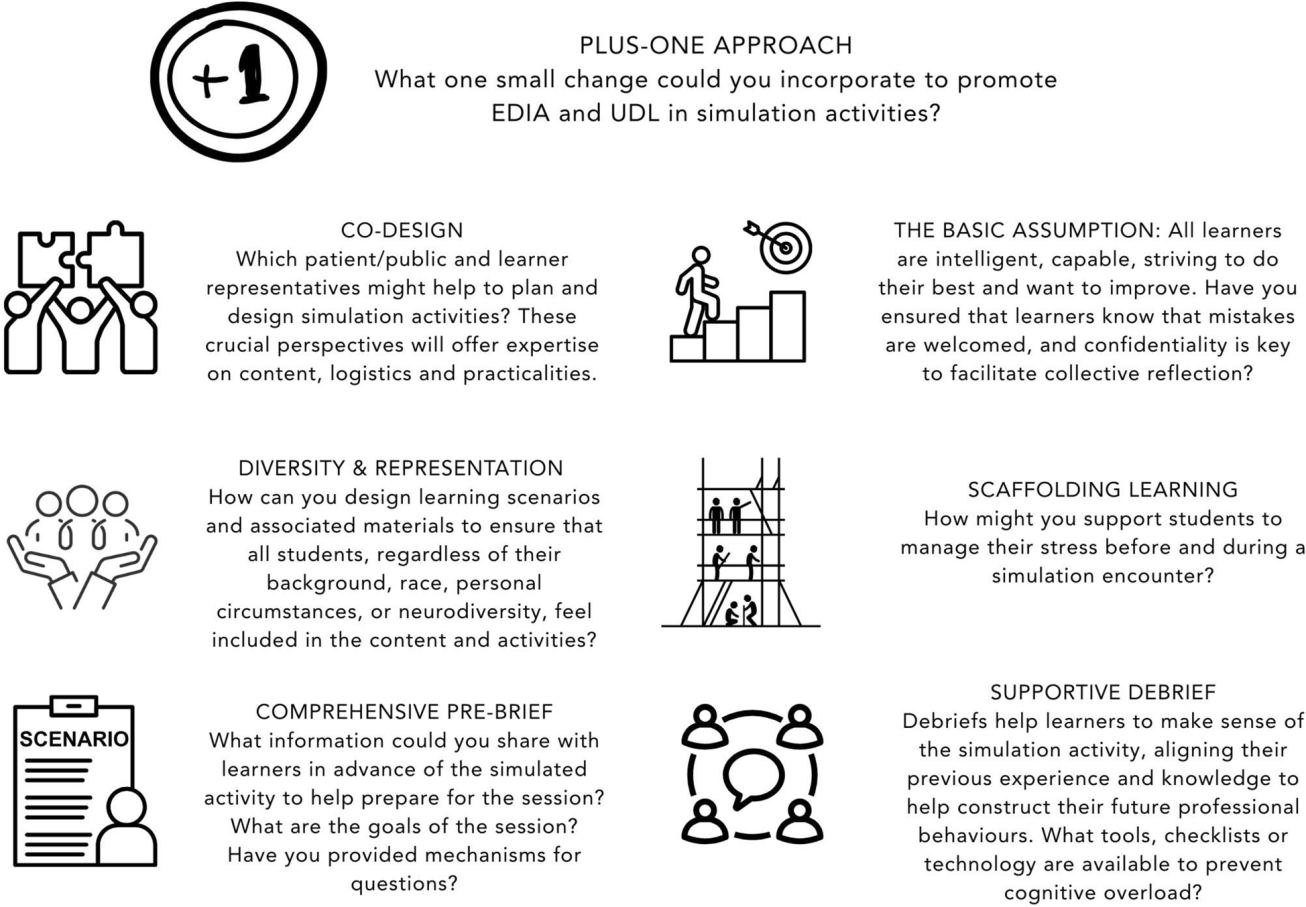
Thought-provoking questions for simulation educators to encourage small incremental changes to curricula as an introduction to the UDL principles

Figure 2 offers some thought-provoking questions for simulation educators to consider and to reflect upon as they consider how they might make a small change or adopt the “Plus One” approach in their activities.

## Conclusion

This article has demonstrated that UDL can be applied as a theoretical framework to underpin best practice simulation-based education. By integrating UDL principles, simulationists can enhance the inclusivity and effectiveness of their learning environments, ensuring that all learners have the opportunity to succeed. To accurately reflect real-world scenarios, it is essential that simulations mirror the diversity of patients and healthcare providers. Embracing UDL principles enables educators to create simulation experiences that meet the diverse needs of learners, fostering an inclusive environment where everyone can thrive. Future research should explore UDL as a theoretical framework for simulation and also the impact of UDL and other curriculum diversification approaches on learning outcomes, learner satisfaction, and organizational performance. Honouring “The Basic Assumption”, we owe it to learners in simulation to adapt to their learning needs. Next time you plan a simulated activity, adopt the Plus One Approach and think about how to include UDL Principles, for the benefit of your learners and their future patients.

## Data Availability

No datasets were generated or analysed during the current study.

## Abbreviations

ADHD: Attention-deficit/hyperactivity disorder
CAST: The Centre for Applied Specialized Technology
CoP: Communities of practice
EDIA: Equity, diversity, inclusivity, and accessibility
PEARLS: Promoting Excellence And Reflective Learning in Simulation
SBE: Simulation-based education
SCT: Social Cognitive Theory
UDL: Universal Design for Learning
UX: User experience
VR: Virtual reality
WHO: World Health Organisation
